# Interferon gamma as an immune modulating adjunct therapy for invasive mucormycosis after severe burn – A case report

**DOI:** 10.3389/fimmu.2022.883638

**Published:** 2022-08-22

**Authors:** Dina M. Tawfik, Caroline Dereux, Jan-Alexis Tremblay, Andre Boibieux, Fabienne Braye, Jean-Baptiste Cazauran, Meja Rabodonirina, Elisabeth Cerrato, Audrey Guichard, Fabienne Venet, Guillaume Monneret, Didier Payen, Anne-Claire Lukaszewicz, Julien Textoris

**Affiliations:** ^1^ “Pathophysiology of Injury-Induced Immunosuppression”, Université Claude Bernard Lyon-1 - Hospices Civils de Lyon - BioMérieux, Lyon, France; ^2^ Open Innovation and Partnerships (OIP), BioMérieux S.A., Lyon, France; ^3^ Anesthesia and Critical Care Department, Hôpital Edouard Herriot, Hospices Civils de Lyon, Lyon, France; ^4^ Critical Care Department, Hôpital Maisonneuve Rosemont, Université de Montréal, Montréal, QC, Canada; ^5^ Service des Maladies Infectieuses et Tropicales, Hôpital de la Croix-Rousse, Hospices Civils de Lyon, Lyon, France; ^6^ Service de Chirurgie Plastique, Reconstructrice et Esthétique, Hôpital de la Croix Rousse, Hospices Civils de Lyon, Lyon, France; ^7^ Service de Parasitologie, Hospices civils de Lyon, Hôpital de la Croix-Rousse, et Université Claude Bernard Lyon 1, Lyon, France; ^8^ Immunology Laboratory, Hôpital Edouard Herriot, Hospices Civils de Lyon, Lyon, France; ^9^ Université Paris-7 Denis Diderot, Paris, France

**Keywords:** Mucormycosis, interferon gamma adjunct therapy, immunotherapy, biomarkers, severe burn injuries

## Abstract

**Background:**

Mucormycosis is a deadly fungal infection that mainly affects severely immunocompromised patients. We report herein the case of a previously immunocompetent adult woman who developed invasive cutaneous mucormycosis after severe burn injuries. Interferon-gamma (IFN-γ) treatment was added after failure of conventional treatment and confirmation of a sustained profound immunodepression. The diagnosis was based on a reduced expression of HLA-DR on monocytes (mHLA-DR), NK lymphopenia and a high proportion of immature neutrophils. The immune-related alterations were longitudinally monitored using panels of immune-related biomarkers.

**Results:**

Initiation of IFN-γ was associated with a rapid clinical improvement and a subsequent healing of mucormycosis infection, with no residual fungi at the surgical wound repair. The serial immunological assessment showed sharp improvements of immune parameters: a rapid recovery of mHLA-DR and of transcriptomic markers for T-cell proliferation. The patient survived and was later discharged from the ICU.

**Conclusion:**

The treatment with recombinant IFN-γ participated to the resolution of a progressively invasive mucormycosis infection, with rapid improvement in immune parameters. In the era of precision medicine in the ICU, availability of comprehensive immune monitoring tools could help guiding management of refractory infections and provide rationale for immune stimulation strategies in these high risk patients.

## Introduction

Mucormycosis infection is a rare, rapidly progressing and often fatal disease caused by the Mucorales fungi (1). This type of infection is opportunistic and affects mainly immunocompromised patients suffering from severe underlying conditions such as hematological disorders, organ transplantation or longstanding diabetes (2).

Classically, management of mucormycosis revolves around early diagnosis, reversal of the underlying immunosuppression, aggressive surgical debridement and antifungal therapy (2). Early initiation of anti-fungal therapy is usually associated with better outcomes, and a lipid formulation of amphotericin B was the drug of choice (5 to 10mg/kg daily). In our burn center, after infectious disease specialist consultation and depending on the clinical course, we sometimes add posaconazole, although there is little data to support such approach. Posaconazole delayed-release tablets is however a preferred molecule in our center to continue the relay of IV treatment. Adjunct immunotherapy such as Interferon-gamma (IFN-γ) can stimulate and modulate the immune system in critically ill patients and has been previously reported as rescue therapy for immunosuppressed patients struggling with persistent fungal infections and sepsis (3, 4). IFN-γ is a cytokine mainly produced by T_H_1 and Natural Killer (NK) cells. It activates circulating monocytes and restores expression of monocytic Major Histocompatibility Class II (MHC II) Human Leukocyte Antigen-DR (mHLA-DR) molecule, thus increasing their antigen-presenting capacity and phagocytic function (5).

Here, we report the case of a critically ill severe burn patient that suffered from an invasive mucormycosis infection that was refractory to multimodal antifungal therapy and was treated with recombinant IFN-γ adjunct immunotherapy. We also describe the clinical and immune alterations in response to IFN-γ therapy using different markers in multiple diagnostic platforms that represent different facets of the immune response.

## Clinical description

**Figure 1:**
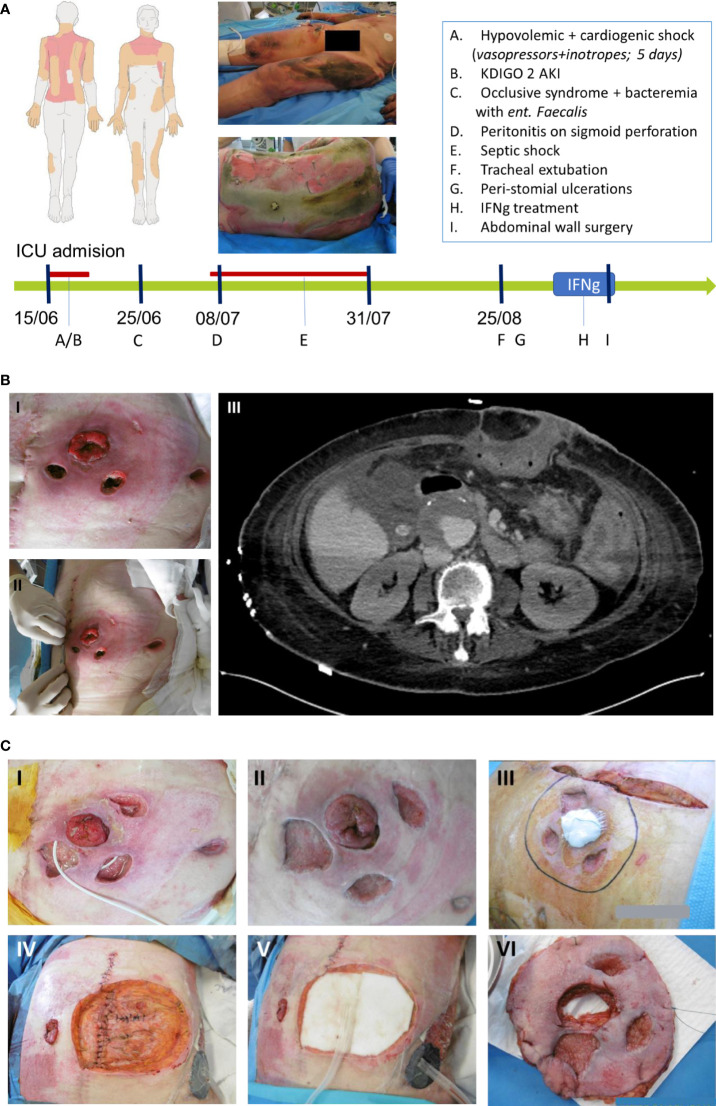
**(A)** Burn map of the burn injuries sustained by the patient, and timeline of events incurred by the case in Intensive Care Unit (ICU) represented by the green arrow while the red line represents the duration of vasopressors administration. **(B)** Mucormycosis of the abdominal wall. **(B.I.)** and **(B.II.)** Show peristomial ulcerations. **(B.III.)** Horizontal abdominal scan assessing the intra-abdominal extension of the cellulitis. **(C)** Evolution and healing of the abdominal lesions in response to IFN-γ treatment. **(C.I.)** Lesions before IFN-γ treatment. **(C.II.)** and **(C.III.)** Lesions after 6 days of IFN-γ and right before surgery. **(C.IV.) (C.V.)** and **(C.VI.)** Excision of the abdominal wall and the transposition of the colostomy.

We report the case of a 61-year-old woman, with a medical history of hypertension, active smoking and bipolar disease, no history of diabetes, who suffered third-degree burns on 43% of her total body surface area (neck, thorax, back and the four limbs) after an accidental fire (**Figure 1A**).

On ICU admission, the patient presented signs of severe hypovolemic shock necessitating generous fluid resuscitation guided by continuous cardiac output monitoring (PiCCO, Getinge). Ten days after admission she developed septic shock from stercoral peritonitis after ischemic colitis related to the initial shock. She received broad-spectrum antimicrobials and underwent subtotal colectomy with colostomy, after which the septic shock resolved. In parallel, the burn injuries were treated over a course of 11 skin grafts surgeries (**Figure 1**).

After one month in the ICU, as the patient had resolved all organ failures and was slowly improving, two necrotic ulcers were noticed adjacent to the colostomy, and a third one on the left flank (**Figures 1B**). The bacteriological and mycological sampling of the abdominal wounds came back positive for *Rhizopus microspores* (both in culture and pan-fungal PCR). A Computerized Tomography (CT) scan was performed to assess the spread of this abdominal fungal cellulitis which did not show any deep visceral extension of the infection (**Figure 1B**). Blood-PCR assays were drawn and performed twice a week and all tested negative for mucormycosis. Systemic antifungal therapy was initiated using intravenous liposomal amphotericin-B (10mg/kg, once per day) on the 35^th^ day post-admission, combined with oral posaconazole 2mg/kg every 6h. In parallel, the lesions were treated with a 100mg/L of amphotericin-B solution (irrigation three times a day as well as wounds dressing with antifungal-soaked gauze pads) (6).

Despite this multimodal therapy, the necrotic skin lesions were non-responsive to treatment and continued to progress over the next month. A multidisciplinary meeting was held to assess the feasibility of abdominal wall resection surgery. Concerns were raised that moving the colostomy on the right side of the abdominal wall may expose the patient to intra-peritoneal contamination with mucormycosis. An immune status review revealed low mHLA-DR expression (Day 83: 7439 Ab/C; Day 88: 7537 Ab/C), significant NK lymphopenia (Day 83: 22 cell/µL; Day 88: 39 cell/µL) and a high proportion of immature neutrophils (CD10**^-^**/CD16**^-^** PMN: 82% of PMN on Day 88) indicative of a profound innate immune response dysfunction (7, 8). At that time, there was no T or B cells lymphopenia observed (Day 88: T-cells: 1029/µL, B-cells: 134/µL). IFN-γ adjunctive immune therapy was thus initiated for 7 days before the abdominal wall resection surgery and the colostomy transposition in efforts to control the infection. The patient received Interferon gamma-1b (IMUKIN^®^ Boehringer-Ingelheim, Germany) at a regimen of 100µg dose, subcutaneously for 7 consecutive days. A slight myalgia and 38°C fever for a few hours were noted after the first and the second injections. The maximal temperature reached was 38.8°C after the 6^th^ injection, she did not develop any major adverse effects and the liver enzymes remained stable without any signs of cytolysis.

The abdominal wall resection surgery was performed on the 7^th^ day of treatment, and the last IFN-γ injection was administered post-operatively. Macroscopically, IFN-γ treatment was associated with rapid signs of microbiological cure (**Figure 1C**), showing clear healing of the previously inflammatory and necrotic lesions. The surgery consisted of a large resection of the abdominal wall (**Figure 1C**), transposition of the colostomy, and vacuum therapy with subsequent skin flap graft on the excised zone. All surgical specimen examination were negative for *Rhizopus* microspores, including histological examination, PCR of tissue biopsy or tissue culture. Histological examination found granular tissue with polymorph inflammatory infiltrates without hyphae either on Periodic Acid-Schiff (PAS) or Grocott coloration tests. Antifungal treatment with amphotericin B and posaconazole was administered for a total of 75 days, with posaconazole monotherapy further continued for a year.

The patient left the ICU after 236 days for a long-term care facility. She was seen as an outpatient thirteen month after the initial ICU admission to assess burn scar evolution and the feasibility of colostomy closure. Skin examination showed complete burn healing with incomplete maturation of the burn scars and no major burn contracture. Physical autonomy was still incomplete, abdominal wall remained very thin and fragile and colostomy closure could not be performed at that time. She was followed up until two years later with no reported complications.

## Immune monitoring

As part of our research interest in the host immune response, we performed a deep assessment of the immune response before, during and after IFN-γ treatment. Besides common laboratory tests, we collected samples to perform flow cytometry (**Supplementary Figure S1**), quantification of protein soluble markers (**Supplementary Table S1**) and transcriptional response (**Supplementary Figures S2, S3** and **Supplementary Tables S2**, **S3**), as well as immune functional assays (IFA) (**Supplementary Table S4**).

**Figure 2:**
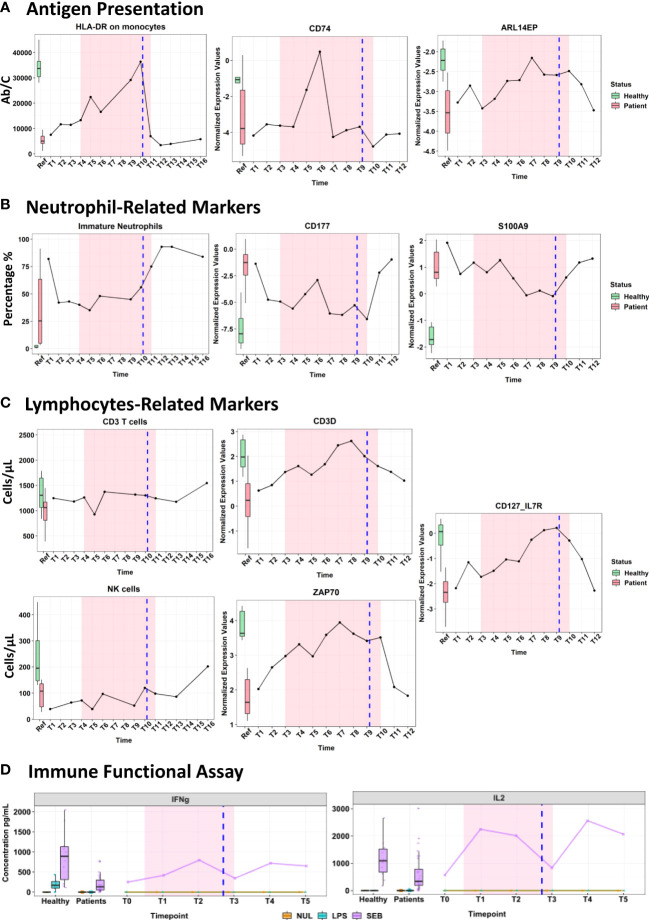
Immunomonitoring of cell surface markers using flow cytometry, transcriptomic analysis using the Immune profiling Panel (IPP) prototype and cytokines measured by ELLA platform after being stimulated in Truculture^®^ tubes (Immune functional assays). Markers’ measurements are represented on the y-axis, while the x-axis indicates the different time points measured. The highlighted pink area denotes the duration of the IFN-γ treatment. The vertical blue line denotes the day of the surgery. The boxplots on the left illustrate expression levels in healthy volunteers (green) and ICU patients (red) as reference. **(A)** Evolution of antigen presentation pathway. **(B)** Neutrophil-related markers. **(C)** Lymphocyte-related markers. **(D)** Cytokine production after immune functional assays.

As expected, treatment with IFN-γ had a strong effect exhibiting a transient activation of the antigen presentation pathway and monocytes-related markers reflected by the 3-fold increased mHLA-DR expression from 11,447 Ab/C to 36,375 Ab/C (measured on the day of the surgery - **Figure 2A**). On the transcriptomic level, an upregulation of CD74 and ARL14EP expression was observed reaching the levels of the healthy reference group (**Figure 2A**). CD74, an HLA-DR antigen-associated invariant chain (9) has a role in MHC class II trafficking to the antigen processing compartments (10), while, ARL14EP controls the export of MHC class II molecules from endosomal MIIC (11).

The overall neutrophil count decreased during treatment, while no impact was observed on the percentage of immature neutrophils (43-56% of CD10**^-^**/CD16; normal value < 5%) until mid-treatment when their proportion started to rise again. Neutrophil cell surface transcriptomic markers: CD177 (neutrophil-specific marker) and S100A9 (a DAMP expressed on both neutrophil and monocyte) (**Figure 2B**) showed a transient downregulation associated with IFN-γ treatment.

A steady delayed increase in total and B lymphocyte counts was observed in the week following IFN-γ treatment. Along with a steep increase in transcriptomic markers: CD3D, ZAP70 and IL-7R (**Figure 2C**) was observed at the initiation of IFN-γ therapy. All three genes are involved in T-cell receptor coding, downstream signal transduction as well lymphocyte homeostasis and survival (12–15). NK cells (which are a key part of anti-fungal immunity) tended to increase under treatment, and this effect was persistent after the surgery (from 64-120 cells/µL on the day of surgery, and 202 cells/µL a week later) (**Figure 2C**).

Two standardized IFA (whole blood stimulation with LPS: Lipopolysaccharide and SEB: Staphylococcus Enterotoxin-B, using standardized TruCulture^®^ tubes) were used to stimulate whole blood for 24 hours (Supplementary Methods) to reveal the immune cell function. After stimulation by LPS and SEB, the plasma cytokine levels of IL-6, TNF and IL-10 were within the expected range for the septic patients, however, no changes were observed during the IFN-γ treatment (**Supplementary Table S4**). Interestingly, the SEB stimulation highlighted a clear immediate increase of IFN-γ (3-fold) and IL-2 (4.5-fold) cytokines that was maintained until the end of IFN-γ therapy (**Figure 2D**). More importantly, after a transient decline due to surgery, the levels of IL2 and IFN-γ cytokines response to stimulation remained high in plasma one week later post-immunotherapy. Both cytokines have a known instrumental function in promoting the development, survival, and proliferation of the innate and adaptive immune cells (monocytes, T-cells, NK, and B cells) (16).

## Discussion

Mucormycosis invasive infection occurs largely in immunosuppressed patients and is associated with catastrophic outcomes, with mortality rates ranging from 70-90% in invasive cases, with surviving patients typically necessitating aggressive and debilitating surgical excision (3). Severe burn patients suffer from frequent secondary infections due in part to loss of skin barrier and exposed open wounds, but also as a consequence of the profound immunosuppression that is associated with the dysregulated host response to initial burn injury (5, 17). Recent guidelines recommend the prompt initiation of high dose liposomal Amphotericin B (10mg/kg) together with surgical debridement as first-line treatment (18). Despite the lack of clinical data on the benefit to use a combined approach of antifungal (e.g. amphotericin + posaconazole or isavuconazole), these drugs are still widely used to provide a broader coverage with a potential synergistic effect (19).

We report the case of a severe burn patient who secondarily developed mucormycosis infection one month after the initial ICU admission. Despite the prompt use of intensive multimodal antifungal therapy as soon as the mucor infection was detected, the necrotic lesions continued to progress. Since immunosuppression is a key factor in the pathophysiology of mucormycosis, reversing immunosuppression through the compassionate use of IFN-γ was proposed as adjunct therapy, as previously reported in other contexts under longitudinal immune monitoring (16, 20). Our extensive immune monitoring revealed the profound acquired immunosuppression and showed signs of immune recovery, as previously reported in other ICU contexts (8). The restored monocyte function and TCR signaling stimulate host defense cytokines to clear persistent fungal infections in non-responsive patients (4, 21). IFN-γ therapy had activated the antigen presentation pathway as reflected by the increased expression of CD74, ARL14EP and mHLA-DR. Furthermore, a slow and delayed recovery was observed in the NK cells count and the lymphocyte subsets (CD4^+^, CD8^+^ T cells as well as CD19^+^ B-cells) after treatment, which persisted 2 weeks later after stopping the INFγ therapy. Previous studies reported a “modest” to no change in the lymphocyte subsets in response to IFN-γ therapy (16, 22). The observed delayed recovery in our case might be multifactorial (1): a predominant effect of INFγ on innate immune response, which consequently activates T Cell Receptor (TCR), and the adaptive immune response (2); an improvement of surviving lymphocytes, as reflected by an upregulation in the lymphocyte proliferation markers (CD3D, CD127 and ZAP70), as well as the increased levels of IFN-γ and IL2 (revealed through IFA). Finally, the transient downregulation of neutrophil subsets and related markers could be a sign of immune recovery as IFN-γ regulates the over-activation of the inflammatory response and tissue damage (23).

In light of the current pandemic, adjunct immunotherapy can have a potential role in improving the outcome of COVID-19 patients. It has been established that COVID-19 patients often suffer from immunosuppression that could be meditated by SARS-CoV-2, corticosteroids or mechanical ventilation during management as well as background co-morbidities (24). Recently, severe COVID-19 infection has also been strongly associated with increased incidence of mucormycosis co-infection, namely in developing countries, further jeopardizing fragile healthcare infrastructures (25). In recent studies, the potentials of IFN-γ immunotherapy to promote immune recovery was demonstrated in COVID-19 patients suffering from SARS-CoV-2 persistence or recurrent ventilator-associated pneumonia. Immune recovery was demonstrated by the increased mHLA-DR expression and lymphocyte counts reflected by improved clinical outcomes such as avoiding mechanical ventilation and restriction of superinfections (24, 26, 27).

This clinical case study underscores the potential of monitoring the immune response of critically ill patients with refractory infections. Nonetheless, only one patient was reported that precludes any generalization. It emphasize the role of such monitoring for personalized therapy in complex situations. Some tested biomarkers in this study require further validation in large cohort of patients to be validated (28). This is reflected in some transcriptional response of biomarkers that fluctuated potentially in relation with biological feedback loops changes in response to therapy and/or possible clinical interventions for the case management.

In conclusion, the treatment approach led to a positive and unequivocal clinical response and surgical exploration showed microbiological cure, with PCR samples all negative for mucor following treatment. In this critically ill patient, IFN-γ immunotherapy was associated with potent signs of innate immune response recovery, long-standing adaptive recovery, and was well-tolerated with no signs of cytokines overshooting. This case emphasizes the pertinence of monitoring the immune response of critically ill patients with refractory infections. In an era of precision-based medicine, it further demonstrates that the availability of bedside immune monitoring tools may help to make a therapeutic decision and to monitor the use of immune adjunct therapy in critical illness.

## Data availability statement

The original contributions presented in the study are included in the article/**Supplementary Material**. Further inquiries can be directed to the corresponding author.

## Ethics statement

Ethical review and approval was not required for the study on human participants in accordance with the local legislation and institutional requirements. The patients/participants provided their written informed consent to participate in this study. Written informed consent was obtained from the individual(s) for the publication of any potentially identifiable images or data included in this article.

## Author contributions

DT, GM, DP, A-CL and JT contributed to design of the study and redaction of the manuscript. CD, J-AT, AB, FB, J-BC, MR, A-CL and JT organized he management of the patient and supervised the clinical decisions. DT and JT performed the statistical analysis. EC, AG, GM and FV performed laboratory analysis and transcriptomics. DT and JT wrote the first draft of the manuscript. FV, GM, DP, A-CL and JT wrote sections of the manuscript. All authors contributed to manuscript revision, read, and approved the submitted version

## Acknowledgments

We would like to acknowledge the nurses at the intensive care unit and equally the technicians at the Immunology department at Edouard Herriot Hospital - Hospices Civils de Lyon for their help in this clinical case report.

## Conflict of interest

Authors JT, EC and AG are employees of an in-vitro diagnostic company (bioMérieux) and hold shares of the company. The company they work for hold patents related to the content of this work. Authors JT, EC, AG, DT, CD, JAT, FV, GM, and ACL are part of a joint research unit co-funded by bioMérieux, Hospices Civils de Lyon and Université Claude Bernard Lyon 1. These entities hold shared patents related to the content of this work.

The remaining authors declare that the research was conducted in the absence of any commercial or financial relationships that could be construed as a potential conflict of interest.

## Publisher’s note

All claims expressed in this article are solely those of the authors and do not necessarily represent those of their affiliated organizations, or those of the publisher, the editors and the reviewers. Any product that may be evaluated in this article, or claim that may be made by its manufacturer, is not guaranteed or endorsed by the publisher.
