# Supplementary material for: Interferon gamma as an immune modulating adjunct therapy for invasive mucormycosis after severe burn – A case report
Source: Front Immunol. 2022 Aug 22;13:883638. doi: 10.3389/fimmu.2022.883638 (PMC9442803; doi:10.3389/fimmu.2022.883638)
Supplement: Supplementary file 1 [file DataSheet_1.docx]

# Supplementary Data

## **Supplementary Methods**

**Blood collection and stimulation using TruCulture System** Whole blood from the burn patient was collected at different time points (longitudinal follow-up). The patient’s status was followed over almost a month using different immune response detection methods. Heparin, EDTA and PAXgene collection tubes were used according to the measurement method. The blood tubes were immediately transferred to the lab and processed within 3h after blood sampling. Blood collected in EDTA tubes was used for flow cytometry immune phenotyping and plasma cytokine level measurements. Blood collected in PAXgene tubes was inverted several times and incubated for 2 hours at room temperature according to the manufacturer’s recommendation. PAXgene-stabilized blood was used for the transcriptomic analysis using the Immune Profiling Panel (IPP) tool prototype.

For the Immune Functional Assays (IFA), 1 mL of heparinized whole blood was distributed into TruCulture tubes (Myriad Rbm, USA) containing media alone (NUL), media with LPS (100 ng/mL) and media with SEB (400 ng/mL). LPS (a component of gram-negative bacteria wall, also known as endotoxin) and SEB (Staphylococcus Enterotoxin-B), a superantigen able to crosslink antigen-presenting cells and induce a polyclonal activation of CD4+ and CD8+ T cells (1). The TruCulture/blood mix was incubated for 24 hours at 37°C in a dry block incubator and the supernatants were retrieved for the soluble markers’ quantification using ELLA platform.

**Immune cell counts and immune cells phenotyping by flow cytometry** Surface markers were assessed on peripheral whole blood collected in EDTA anticoagulant tubes using flow cytometry (NAVIOS; Beckman-Coulter, USA) as previously published (2,3). The assessed surface markers included measurement of mHLA-DR expression (number of antibodies bound per cells (AB/C), percentage of CD16^low^ CD10^low^ neutrophils among total circulating neutrophils and number of circulating T lymphocytes, monocytes, natural killer cells (cells per µl of blood), CD3^+^, CD4^+^ and CD8^+^ T-cells, CD19^+^ B-cells as well as the percentage of PD-1 expressed on CD4^+^and PD-1 expressed on CD8^+^ cells.

**Protein detection** Soluble cytokine markers in plasma from the supernatant collected from the EDTA tubes and after stimulation in TruCulture tubes were quantified using ELLA nanofluidic system (Biotechne, Minneapolis, MI, USA), according to the manufacturers’ instructions. The soluble cytokine markers measured included TNF, IL-10, IL-6, IFN-γ and IL-2. Results were expressed in pg/ml.

**Transcriptomic analysis**

**Immune profiling Panel (IPP)** Whole blood samples (PAXgene) were tested using the IPP tool prototype according to the manufacturer’s instructions. Briefly, the pouches were hydrated with the hydration solution supplied with the kit. A 100 μL of either PAXgene or TruCulture mix was mixed with approximately 800 μL of the lysis buffer provided with the kit and directly injected into the pouch and ran on FilmArray 2.0 and FilmArray Torch instruments (BioFire, Inc., Salt Lake City, UT, USA). Results were delivered as normalized expression values of markers (4). IPP is a multiplex PCR prototype designed in our laboratory, based on the FilmArray system which is an integrated platform that allows automated nucleic acid extraction and multiplex nested PCR amplification in 1 hour (4).

**Nanostring platform** On-column RNA extraction was performed on the cell pellet collected after stimulation and the transcriptomic immune response was evaluated using an in-house 86-gene panel using NanoString technology as previously published (5). Data was normalized using nSolver analysis software (version 4.0, NanoString technologies) and results were expressed in counts. Gene expression data that did not pass the quality control analysis were not included (Supplementary Figure S2).

**Reference healthy volunteers and patients** Retrospective blood samples from healthy volunteers and patients were obtained from the REALISM cohort tested using the IPP tool. REALISM cohort is a prospective, single-center, observational trial that included critically ill patients admitted to the ICU at Édouard Herriot hospital (Hospices Civils de Lyon, France) clinicaltrials.gov (NCT02803346) (6). Reference data for the immune functional assays (soluble cytokine markers) were obtained from studies performed previously at our laboratory (5).

**Statistical analyses** Graphs and statistical analyses were performed using R (version 3.6.2) (7). Healthy volunteers and patients’ reference results were expressed as median and interquartile ranges [IQR]. Heatmap representation of the transcriptomic data quantified by the Nanostring panel was performed on normalized counts and divided by the sample’s level of expression to adjust for varying basal levels of expression and to reflect the main modulation direction. Rows (markers) were reordered and clustered based on Euclidian distance. Pearson correlation analysis was performed between the common markers in the IPP tool and the Nanostring panel to validate the transcriptome analysis to validate the results obtained by IPP (Supplementary Table S2).

## **Supplementary Results**


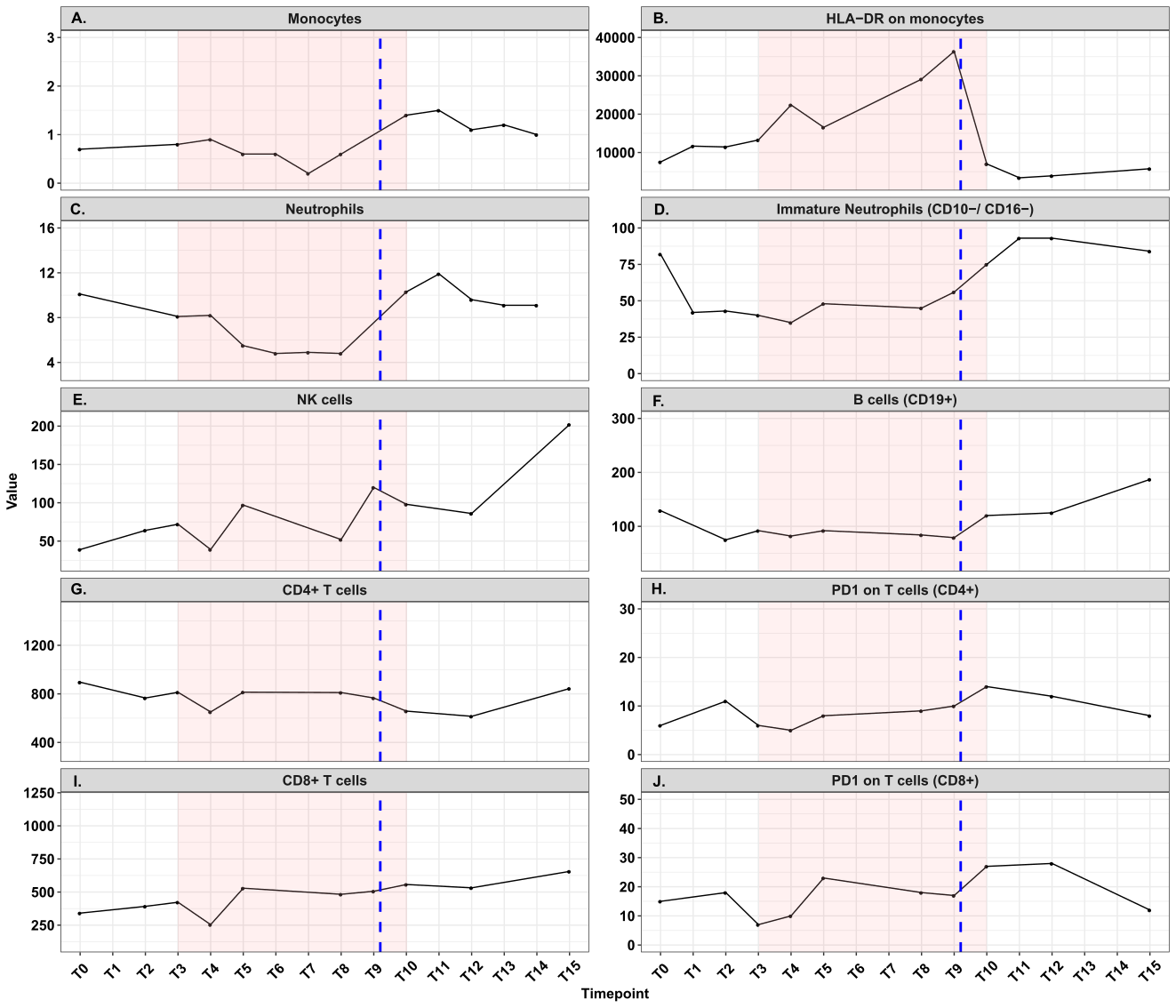


**Supplementary Figure S1** Immune cell counts, and phenotype measured using flow cytometry, y-axis: values of each marker measured, x-axis: time points measured around IFN-γ therapy. The highlighted pink area between “T3” and “T9” denotes the duration of the IFN-γ treatment, and the vertical blue line denotes the day of the abdominal wall surgery. The patient did not present signs of: monocytopenia or B-cell lymphopenia (normal references values in our laboratory: monocytes 0.20-0.90 cells/μL, B cells 224-581 cells/µL,). Before IFN-γ treatment at Timepoint “T2” a decreased mHLA-DR expression (11 447 Ab/C; normal value >20 000 Ab/C) was observed, severe NK lymphopenia (64 cells/µL; normal value 77-321 cells/µL) as well as high proportion of immature (CD10-/CD16-) neutrophils (40%; normal value < 5%), indicative of a profound innate immune response dysfunction.

| Cytokine (pg/mL) | Normal range | Day 94 | Day 95 | Day 96 | Day 97 | Day 98 | Day 101 | Day 102 | Day 103 | Day 104 |
| --- | --- | --- | --- | --- | --- | --- | --- | --- | --- | --- |
| TNF | [1-33] | 30 | 25 | 27 | 23 | 31 | 27 | 31 | 22 | 33 |
| IL-6 | [1-4] | 19 | 22 | 17 | 20 | 22 | 16 | **32** | **438** | **195** |
| IL-10 | [2-4] | 14 | 14 | 16 | 12 | 12 | 12 | 14 | 13 | 15 |
| IL-2 | [9-16] | 1,3 | 1,1 | nd | nd | nd | nd | nd | nd | 1,24 |
| IFNg |  | nd | nd | nd | nd | nd | 5,3 | 59,8 | 7,9 | nd |

**Supplementary Table S1**: Plasma levels of cytokines assessed by ELLA platform; the highlighted pink area represents the duration of treatment. (nd: not detected), normal reference ranges are based on previous studies in our laboratory.


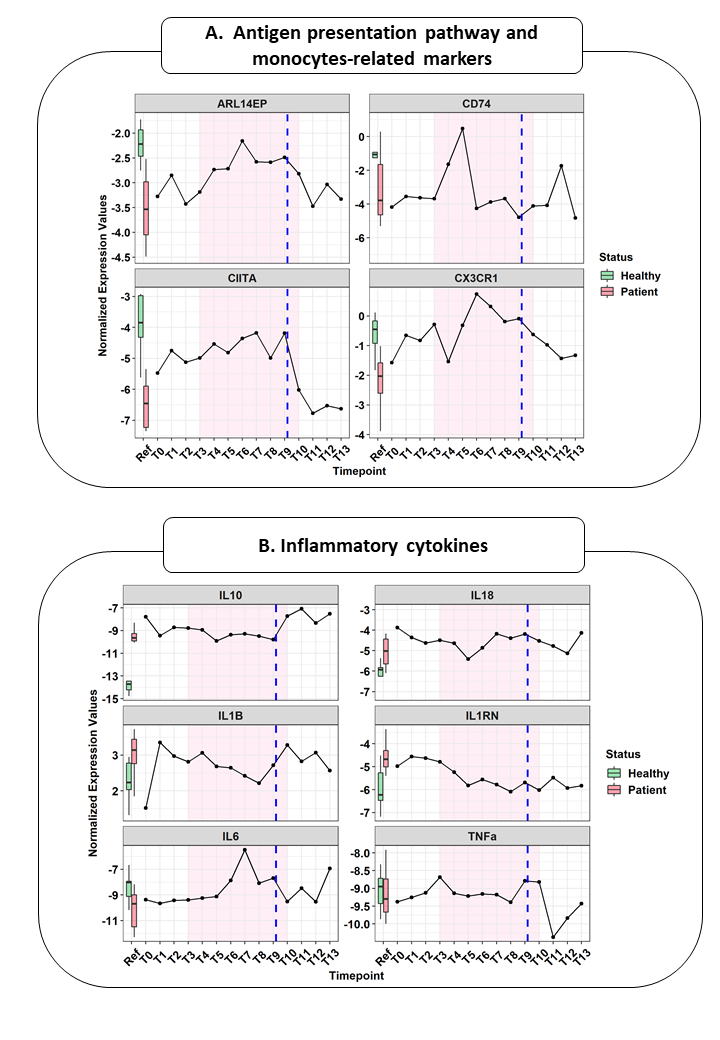

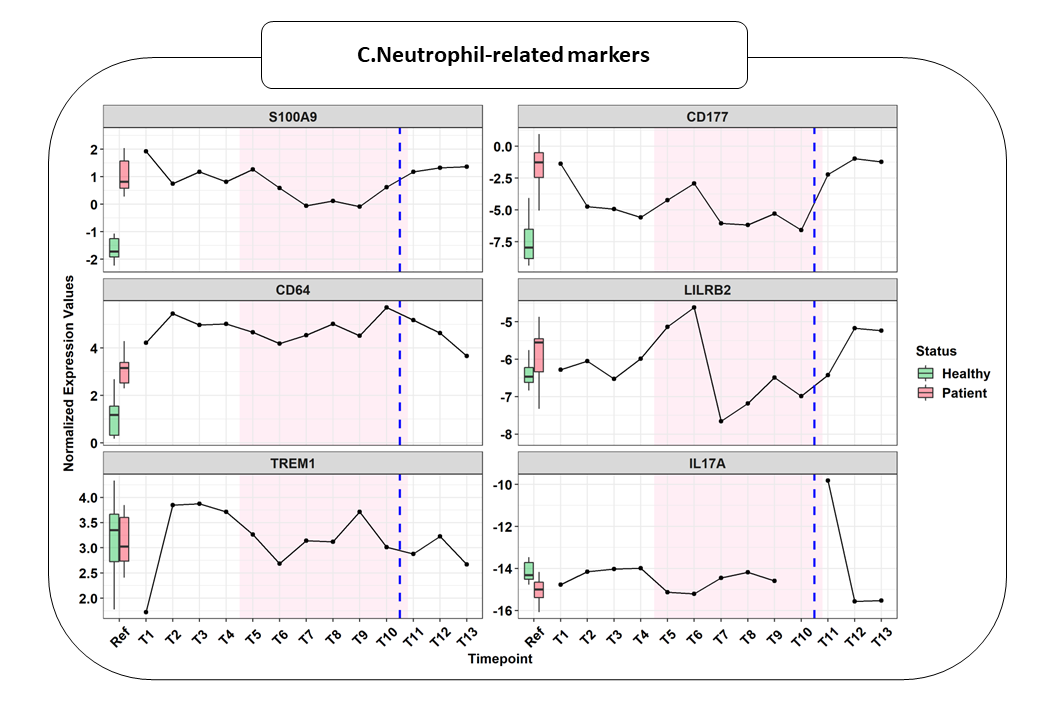

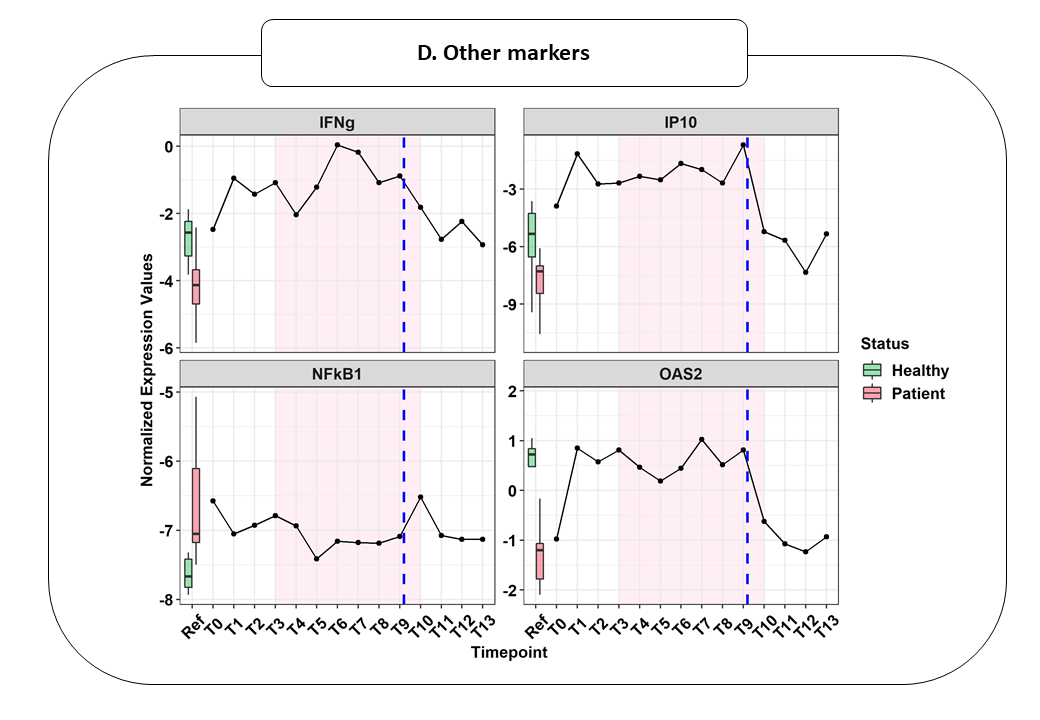

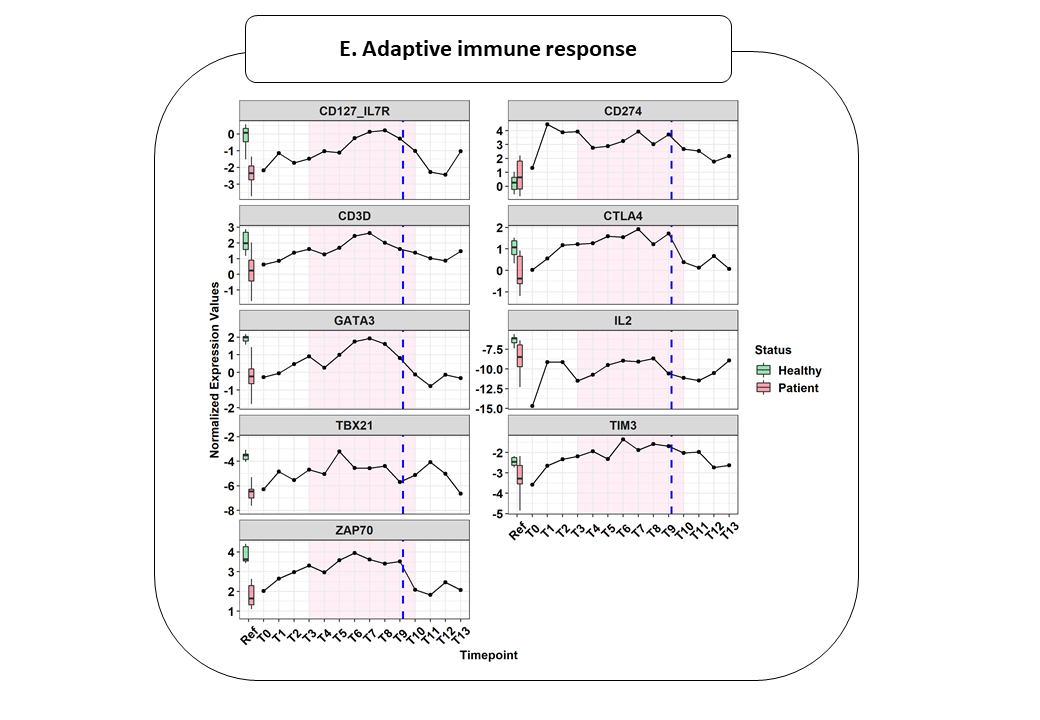


**Supplementary Figure S2** Evolution of transcriptomic markers measured using IPP. The y-axis shows the normalized expression levels, while the x-axis shows the different time points measured. The highlighted pink area denotes the duration of the IFN-γ treatment. The vertical blue line denotes the day of the abdominal wall surgery. The boxplots on the left illustrate expression levels in healthy volunteers (green) and critically-ill patients (red). A. Markers related to antigen presentation pathway and monocyte-related markers. B. Pro-inflammatory and anti-inflammatory cytokine transcriptomic markers. C. Neutrophil-related markers. D. Markers related to IFN- γ response. E. Markers related to the adaptive immune response.


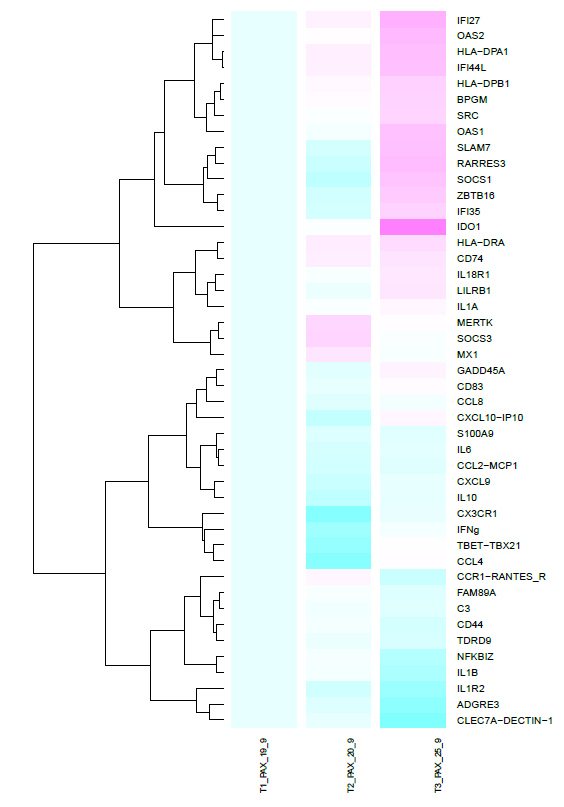


**Supplementary Figure S3** Heatmap representation of the most variable genes quantified by the Nanostring panel. Counts were normalized, and divided by first sample level of expression, to adjust for varying basal levels of expression, and better reflect the main modulation direction. Rows (i.e. genes) were reordered and clustered based on Euclidian distance. Expression levels are color coded from Cyan(down-modulation) to white to Magenta (up-modulation).

| Gene | Pearson correlation coefficient |
| --- | --- |
| ADGRE3 | 0.879 |
| ARL14EP | -0.412 |
| CCNB1IP1 | -0.578 |
| CD3D | 0.551 |
| CD74 | 0.115 |
| CX3CR1 | 0.975 |
| IFNγ | 0.852 |
| IL10 | 0.653 |
| IL18 | 0.458 |
| IL1B | 0.992 |
| IL2 | 0.689 |
| IL6 | -0.503 |
| IP10 | 0.965 |
| MDC1 | 0.757 |
| NFkB | 0.705 |
| OAS2 | 0.430 |
| S100A9 | 0.291 |
| TBX21 | -0.546 |
| TDRD9 | 0.899 |
| TNF | -0.356 |

**Supplementary Table S2** Correlation coefficient (Pearson) for genes common between the two transcriptomic platforms (Nanostring and the Immune Profiling Panel). Genes with good correlation (>0.4) are in green. Negative coefficients are in red.

| Gene | Max variation from baseline | Mean variation from baseline |
| --- | --- | --- |
| ADGRE3 | -2,16 | -1,77 |
| ALOX5 | -2,08 | -1,69 |
| ARL14EP | 1,80 | 1,36 |
| CCNB1IP1 | 1,72 | 1,36 |
| CD127_IL7R | 2,68 | 1,86 |
| CD177 | 8,29 | 1,13 |
| CD274 | -2,26 | -1,81 |
| CD3D | 1,51 | 1,09 |
| CD64 | -1,94 | -1,35 |
| CD74 | 23,44 | 1,44 |
| CIITA | 1,47 | 1,21 |
| CTLA4 | 1,68 | 1,09 |
| CX3CR1 | -2,34 | -1,06 |
| FAS | 1,31 | 1,04 |
| FLT1 | 1,89 | 1,26 |
| GATA3 | 1,51 | 1,08 |
| GNLY | -1,54 | -1,09 |
| GSN | -2,23 | -1,45 |
| HIF1a | -1,53 | -1,25 |
| IFNγ | -1,90 | -1,00 |
| IL10 | -2,66 | -1,83 |
| IL18 | -1,81 | -1,25 |
| IL1B | -1,84 | -1,31 |
| IL1RN | -2,99 | -2,15 |
| IL2 | 5,74 | 3,24 |
| IL6 | 11,29 | 2,43 |
| IP10 | 3,00 | 1,43 |
| LILRB2 | -4,47 | -1,47 |
| MDC1 | -1,90 | -1,47 |
| NFkB | -1,81 | -1,49 |
| OAS2 | -1,81 | -1,35 |
| RORgt | 2,87 | 1,36 |
| S100A9 | -2,56 | -1,52 |
| TBX21 | 3,61 | -1,06 |
| TDRD9 | -1,65 | -1,19 |
| TIM3 | 1,27 | 1,14 |
| TNFa | -1,97 | -1,57 |
| TREM1 | -2,16 | -1,69 |
| ZAP70 | 1,57 | -1,00 |

**Supplementary Table S3** Mean and max variation (ratio from baseline) during IFN-γ treatment (excluding the time point post-surgery). Up-modulated are in red, and down-modulation are in blue (threshold |1.5)

**A.**

|  | Healthy | | | Patients | | |
| --- | --- | --- | --- | --- | --- | --- |
| Cytokine pg/mL | **NUL** | **LPS** | **SEB** | **NUL** | **LPS** | **SEB** |
| IL2 | 0  (0-0.2) | 0  (0-0.3) | 1092.3  (677-1524.2) | 0.4  (0.2-0.7) | 0.1  (0-0.2) | 338  (190-784.2) |
| TNF | 1.8  (1.7-2.1) | **5216.3**  (4204.4-5711.4) | 754.3  (517.7-832.4) | 6  (3.7-7.1) | **705.9**  (336.2-1317.6) | 238.3  (126.6-418.2) |
| IFN-ƴ | 0  (0-0) | 171.4  (98.5-262.9) | 890.9  (311.3-1131.3) | 0  (0-0.1) | 0  (0-0) | 131.6  (53.6-297.2) |
| IL6 | 1.2  (0.7-1.7) | 31415.3  (25511.4-38190.8) | 1481  (266.4-3132.4) | 23.7  (12.3-64.1) | 6577.7  (2470.1-12422) | 126.9  (61.5-649.1) |
| IL10 | 0.6  (0.4-0.7) | 367.1  (143-425.7) | 160.8  (123-196.7) | 5  (3.5-12.2) | 92.8  (49.2-157.1) | 39.7  (24-55.5) |

| Cytokine pg/mL | Stimulation | Timepoints | | | | | | |
| --- | --- | --- | --- | --- | --- | --- | --- | --- |
|  |  | **T0** | **T1** | **T2** | **T3** | **T4** | **T5** | **T6** |
| IL2 | NUL | - | - | - | - | - | - | - |
|  | **LPS** | - | - | - | - | - | - | - |
|  | SEB | 569,1 | 2249,7 | 2015 | 831,7 | **2561,1** | 2070,0 | 569,1 |
| TNF | **NUL** | 6,287 | 6,091 | 6,026 | 6,332 | 7,246 | 7,166 | 5,632 |
|  | LPS | 996,199 | 6,035 | 1068,5 | 1019,6 | 643,31 | 555,864 | 648,16 |
|  | **SEB** | 106,95 | 527,74 | 669,16 | 111,69 | 448,73 | 355,129 | 106,95 |
| IFN-ƴ | NUL | - | - | - | - | - | - | - |
|  | **LPS** | - | 236,138 | - | - | - | - | - |
|  | SEB | 249,39 | 416,43 | **796,71** | 345,52 | 720,21 | 648,864 | 249,39 |
| IL6 | **NUL** | 6,628 | 11,133 | 6,228 | 4,658 | 117,48 | 33,168 | 11,479 |
|  | LPS | - | 6,666 | 5195,7 | 4498,7 | 4988 | 4800,914 | 4752,3 |
|  | **SEB** | 127,37 | 441,11 | 481,5 | 237,32 | 945,3 | 85,01 | 127,37 |
| IL10 | NUL | 3,592 | 7,193 | 3,35 | 3,46 | 5,602 | 3,308 | 3,03 |
|  | **LPS** | 67,922 | 4,5155 | 65,679 | 68,914 | 68,179 | 79,008 | 72,211 |
|  | SEB | 13,039 | 52,623 | 47,993 | 22,246 | 40,048 | 21,978 | 13,039 |

**Supplementary Table S4** Assessing the changes of the cytokine profile in plasma around the IFN-ƴ treatment after stimulation using Immune functional assays in TruCulture tubes. **A.** Plasma reference values of cytokines difference between healthy volunteers and patient’s population in the ICU. The values are represented as median and interquartile range. **B.** Assessment of the cytokine profile after stimulation at different timepoints. The highlighted pink area represents the duration of IFN-ƴ therapy.

**References**

1. Petersson K, Pettersson H, Skartved NJ, Walse B, Forsberg G. Staphylococcal enterotoxin H induces V alpha-specific expansion of T cells. J Immunol. 2003 Apr 1;170(8):4148–54.

2. Döcke WD, Höflich C, Davis KA, Röttgers K, Meisel C, Kiefer P, et al. Monitoring temporary immunodepression by flow cytometric measurement of monocytic HLA-DR expression: a multicenter standardized study. Clin Chem. 2005 Dec;51(12):2341–7.

3. Venet F, Chung CS, Kherouf H, Geeraert A, Malcus C, Poitevin F, et al. Increased circulating regulatory T cells (CD4+CD25+CD127−) contribute to lymphocyte anergy in septic shock patients. 2010;16.

4. Tawfik DM, Vachot L, Bocquet A, Venet F, Rimmelé T, Monneret G, et al. Immune Profiling Panel: A Proof-of-Concept Study of a New Multiplex Molecular Tool to Assess the Immune Status of Critically Ill Patients. The Journal of Infectious Diseases. 2020 Jul 21;222(Supplement_2):S84–95.

5. Albert Vega C, Oriol G, Bartolo F, Lopez J, Pachot A, Rimmelé T, et al. Deciphering heterogeneity of septic shock patients using immune functional assays: a proof of concept study. Scientific Reports. 2020 Sep 30;10(1):16136.

6. Rol ML, Venet F, Rimmele T, Moucadel V, Cortez P, Quemeneur L, et al. The REAnimation Low Immune Status Markers (REALISM) project: a protocol for broad characterisation and follow-up of injury-induced immunosuppression in intensive care unit (ICU) critically ill patients. BMJ Open. 2017 21;7(6):e015734.

7. R Core Team. R: A language and environment for statistical computing. [Internet]. Vienna, Austria: R Foundation for Statistical Computing; 2019. Available from: 2018). https://www.R-project.org/.
